# Goal Setting and Treatment Adherence among Patients with Chronic Illness and Depressive Symptoms: Applying a Patient-Centered Approach

**DOI:** 10.5539/gjhs.v8n6p128

**Published:** 2015-10-26

**Authors:** Eric Houston, Alexander K. Tatum, Arryn Guy, Cassandra Mikrut, Wren Yoder

**Affiliations:** 1 Department of Psychology, Illinois Institute of Technology, Chicago, IL; 2 Department of Counseling Psychology, Loyola University Chicago, Chicago, IL

**Keywords:** adherence, depression, chronic illness, HIV, goal setting, multidimensional scaling

## Abstract

**Objective:**

Poor treatment adherence is a major problem among individuals with chronic illness. Research indicates that adherence is worsened when accompanied by depressive symptoms. In this preliminary study, we aimed to describe how a patient-centered approach could be employed to aid patients with depressive symptoms in following their treatment regimens.

**Methods:**

The sample consisted of 14 patients undergoing antiretroviral therapy (ART) for HIV who reported clinically-significant depressive symptoms. Participant ratings of 23 treatment-related statements were examined using two assessment and analytic techniques. Interviews were conducted with participants to determine their views of information based on the technique.

**Results:**

Results indicate that while participants with optimal adherence focused on views of treatment associated with side effects to a greater extent than participants with poor adherence, they tended to relate these side effects to sources of intrinsic motivation.

**Conclusion:**

The study provides examples of how practitioners could employ the assessment techniques outlined to better understand how patients think about treatment and aid them in effectively framing their health-related goals.

## 1. Introduction

Depression is common among people with chronic illness, adversely affecting their ability to consistently engage in goal-directed activities related to treatment, including consistent adherence to medication regimens (DiMatteo, Lepper, & Croghan, 2000; Grenard et al., 2011; Pangalila et al., 2015; Stanners & Barton, 2012). A large body of research indicates that cognitive biases play a major role in inconsistent goal pursuit among individuals experiencing depression (Joormann & D’Avanzato, 2010). While treatment goals and the behaviors related to them could be thought of or framed in numerous ways, symptoms of major depression may increase accessibility to maladaptive thoughts, hamper one’s ability to disengage from cognitions associated with treatment avoidance, and weaken awareness of alternative ways of framing behaviors (Israel, White, & Gervino, 2015).

Several theoretical models in psychology, such as action identification theory (Vallacher & Wegner, 1985), posit that behaviors can be viewed or mentally represented within a hierarchical model ranging from abstract to concrete. Abstract descriptions of behavior relate to the meaning attached to a given behavior, describing why one is performing it or what purpose the act serves. In contrast, concrete descriptions relate to the mechanics of a behavior and specify how one carries it out. Thus, for a patient following an antiretroviral regimen for HIV, an abstract view of their treatment goal may be “improving my immune system” whereas a concrete view would be “swallowing a pill.” Goal-directed behaviors may also be framed in terms of the extent to which they are related to intrinsic or extrinsic motivation based on self-determination theory (Deci & Ryan, 1985). In addition, goals may be framed by individuals based on the degree to which they are positively or negatively valenced, or in terms based on the extent to which they spur approach versus avoidance-oriented behavior. Much research indicates that the way a goal-related behavior is framed affects the likelihood that it will be pursued (Dweck & Grant, 2008).

Patients experiencing depressive symptoms often have difficulty accessing and identifying ways they frame their treatment goal behaviors when these frames actually facilitate adherence (Watkins, 2011). Depressive symptoms weaken such abilities, partly due to poor concentration, diminished reasoning and problem-solving skills, as well as an impaired ability to flexibly shift attention to cognitions that may aid treatment goal initiation and pursuit. Research indicates, for example, that people experiencing depression and other types of psychological distress experience dysregulation in their ability to flexibly shift the framing of their goal-directed behaviors in a manner that meets the demands of a given situation and facilitates goal attainment (e.g., Cribb, Moulds & Carter, 2006; Lemogne et al., 2006). In the case of behaviors related to the treatment adherence goal, this may mean shifting from a focus on cognitions biased toward the abstract level with a negative valence to those that are concrete or that connect the treatment goal with a larger network of goals and sources of intrinsic motivation. A major challenge facing practitioners working with patients with chronic illness involves aiding them in addressing the negative effects of psychological distress on cognitive processing so that they are able to consistently follow their treatment goals over the long term.

### 1.1 Goal Setting: A Patient-Centered Approach

Shah and Kruglanski (2008) recently introduced a structural dynamics approach that highlights how an individual’s set of working goals, or working goal system, is embedded in a broader context of personal needs, environmental demands, and goal hierarchies. The goals within this working system are intricately intertwined and affected by the constraints and exigencies of the moment. In addition, the manner in which goals are framed by individuals may change based on perceived progress within a broader mapping of their overall goals, demands, and needs. A structural dynamics perspective toward goals proposes that helping patients gain awareness of and learn to strategically utilize the multiple meanings that they could attach to a goal-related behavior is critical to self-regulation over time. Such a patient-centered perspective may be particularly valuable to patients who due to depressive symptoms experience impaired cognitive processes, including a bias toward negatively distorted cognitions (Roiser, Elliott, & Sahakian, 2012).

The purpose of the current study was to describe how two assessment and analytic techniques, paired comparisons and multidimensional scaling (Kruskal & Wish, 1978; Stalans, 1995), could be employed to facilitate a structural dynamics approach to health goal setting. By mapping an individual patient’s treatment-related cognitions within the broader context of his or her goals and needs, these techniques may also be used to bolster other patient-centered clinical interventions aimed at improving adherence. We focused on the structural dynamics approach because it directly addresses how goals are not static representations of an individual’s mentally-envisioned future state. Instead, goals may have multiple meanings attached to them and are influenced by the individual’s other goals and needs. Paired comparisons and multidimensional scaling (MDS) could be used to develop assessments of these meanings as reflected by the way patients think about or describe their treatment goals.

### 1.2 Paired Comparisons and Multidimensional Scaling

In paired comparisons, individuals rate the similarity or dissimilarity of two different items based on their own implicit criteria. MDS, a data analytic tool available through SPSS, uses these ratings to generate a geometric mapping that visually depicts the underlying structure of how a patient’s thoughts and appraisals of treatment influence treatment-related behaviors. Patients experiencing psychological distress may have particular difficulty in identifying how their cognitive processes or ways of framing treatment either facilitate or impede adherence. Paired comparisons and multidimensional scaling could serve as tools that aid patients in developing awareness of these underlying patterns and directing their attention toward cognitive processes that strengthen goal pursuit.

Using data from a previous study of patients undergoing antiretroviral therapy for HIV (Houston, McKirnan, Cervone, Johnson, & Sanfort, 2011), we explored how paired comparisons and MDS could be used in clinical work involving patients with chronic illness who experience symptoms of major depression. Depression has been cited in numerous studies as a factor that increases risk for poor treatment outcomes (Gonzalez, Batchelder, Psaros, & Safren, 2011; Sherr, Clucas, Harding, Sibley, & Catalan, 2011; Springer, Dushaj, & Azar, 2012; Wagner et al., 2011). In addition, participants represented two population groups that experience high levels of psychosocial stressors and continued disparities in HIV treatment outcomes: men who have sex with men and ethnic minorities with low socioeconomic status. Guided by action identification theory and self-determination theory, we thus explored a practical application of paired comparisons techniques and MDS in conjunction with a structural dynamics approach to health-goal setting among patients vulnerable to poor outcomes for HIV treatment.

## 2. Method

### 2.1 Study Population

Data for this exploratory report were derived from a cross-sectional study that focused on the relationship between adherence and treatment-related cognitions among patients undergoing antiretroviral therapy (Houston, McKirnan, Cervone, Johnson, & Sandfort, 2011). In the larger study, 39 male patients were enrolled after being recruited through referrals from health providers and social workers at hospitals, clinics, and community health centers in the Chicago metropolitan area. Participants in the current study were drawn from patients in the larger study who reported clinically-significant depressive symptoms (n=19; 49%). Of those participants, data from five were excluded due to errors during the paired comparisons procedure that would have generated uninterpretable MDS configurations. Thus, the analyses in the current study are based on a subsample of 14 participants.

### 2.2 Procedure

Study sessions were conducted at a community-based health clinic in Chicago that provides services primarily to patients who self-identify as gay, bisexual, or men who have sex with men (MSM). Participants completed study measures in a private room subsequent to being administered informed consent. After completing the study session, participants received a #40 incentive. All procedures for recruitment, data collection, and confidentiality were reviewed and approved by the Institutional Review Boards of the University of Illinois at Chicago and the Howard Brown Health Center.

### 2.3 Measures

#### 2.3.1 Demographics

Participants provided basic demographic data by completing a 12-item questionnaire designed to obtain information such as age, race/ethnicity, education, employment and income.

#### 2.3.2 Depressive Symptoms

Depressive symptoms were assessed using the Center for Epidemiological Studies’ Depression Scale (CES-D; Radloff, 1977). The CES-D is a 20-item measure that has been widely employed in HIV and other health research as a population-oriented depression measure (Kilbourne et al., 2001; Reisner et al., 2010). Using a four-point Likert-type scale, participants were asked to report the frequency of depressive symptoms they experienced during the previous week. Items on the measure represent various components of depressive symptoms, including depressive mood, psychomotor retardation, changes in appetite and sleep, and feelings of guilt, helplessness, and hopelessness. Scores could range from 0 to 60, with higher scores representing greater depressive symptoms. Scores of 16 or greater on the CES-D have traditionally been used as an indicator of clinically significant depression (McDowell & Newell, 1996; Pandya, Metz, & Patten, 2005).

#### 2.3.3 Adherence

Self-reported adherence was assessed with the AIDS Clinical Trials Group (ACTG) adherence questionnaire (Chesney et al., 2000). Participants were asked to indicate within a four-day period the number of doses missed for each medication prescribed, the number of days all doses were missed, and how closely the prescription was followed. The total number of pills missed was subtracted from the number of pills prescribed during the four-day period to obtain an estimate of the number taken. This number was then converted into a percentage to obtain an adherence rate. Adherence rates of 95% and greater are widely believed to be optimal for virological control and immune functioning (Bartlett, 2002); thus, we used the 95% threshold to define optimal adherence. Participants with adherence rates below 95% were considered to have suboptimal adherence. Participants were also asked to provide information regarding the length of their treatment and the extent to which they followed daily dosing schedules and special instructions, such as fluid and dietary intake requirements.

#### 2.3.4 Treatment and Personal Goals

Participants in this study were asked to rate 23 statements which primarily represented ways of describing treatment-related goals as well as other personal desires, needs, and demands. The statements were derived during preliminary research from focus groups and pilot testing with men undergoing antiretroviral therapy (Houston, McKirnan, Cervone, Johnson, & Sandfort, 2011). As Table 1 shows, 14 of these statements, or stimulus items, consisted of basic descriptions of behaviors related to treatment goals (e.g., “opening a bottle of medicine,” “swallowing the medicine,” and “following doctor’s orders”).

**Table 1:** Treatment goal and conceptual marker descriptions with labels used in MDS configurations


Treatment descriptions

Concrete:
Opening a bottle of medicine (open)
Taking medicine with water (water)
Swallowing the medicine (swallow)
Checking the pills (check)
Abstract:
Saving my life (savelife)
Following doctor’s orders (doctor)
Taking medicine for family, partner, or someone else in my life (for_others)
Bettering my immune system (immune)
Being responsible (respnble)
Doing something so that I can live healthier (healthy)
Keeping my life on track (on_track)
Side effects:
Feeling nauseous/vomiting (vomit)
Having diarrhea (diarrhea)
Having problems with sleep (sleep)

Conceptual markers and self-referent item

Something that is positive or pleasant to me (positive)
Something that is negative or unpleasant to me (negative)
Something I choose to do in order to get what I really want (intrinsic)
Something I do because it’s required or someone else wants me to (extrinsic)
A longer-term goal or result of following my HIV treatment plan (abstract)
A little thing I do to follow my HIV treatment plan (concrete)
A thought that makes me want to avoid my HIV treatment plan (avoid)
A thought that makes me want to stick with my HIV treatment plan (approach)
Describes the way I usually think about my HIV treatment plan (me)

To aid in understanding how participants perceived the underlying relationship between treatment goals and other goals or behaviors, we included eight additional items representing conceptual or theoretically-based ways of framing behaviors. Conceptual items included markers for intrinsic and extrinsic motivation, such as “Something I choose to do…” and “Something I do because it is required…” We also included markers for approach or avoidance motives (“…makes me want to stick with my HIV treatment plan” or “…makes me want to avoid my HIV treatment plan”), behaviors that were perceived as positively or negatively valenced, and treatment behaviors identified as concrete or abstract. In the current study, we examined the extent to which participants viewed their treatment goals as related to each of these theoretically-based ways of framing behavior. Thus, the paired comparisons procedure used in this study allowed us to assess the extent to which participants considered the relationship between treatment goals and other goals or activities that were, for example, high with regard to intrinsic motivation or positive valence. Participants could make these comparisons and assign ratings based on nonconscious, individual-specific criteria (Darcy, Lee, & Tracey, 2004). Finally, to determine how salient a given treatment-related cognition was in the mind of a participant, we included a self-referent stimulus item (“…describes the way I usually think about my HIV treatment plan.”). Participants used a seven-point rating scale ranging from “not at all” (1) to “very” (7) to rate the similarity between all possible pairings of the 23 items, which resulted in 253 comparisons. The rating procedure generally took between 45 minutes to one hour. All comparisons were presented in random order by an automated, computer-based survey program; participants responded via the computer, in a private setting.

### 2.4 Data Analytic Strategy

Data analysis was performed using SPSS version 21 (IBM, Inc., Chicago IL). We divided the sample into two groups based on adherence rates (optimal vs. suboptimal). We grouped treatment descriptions into two categories, concrete and abstract, and then calculated the mean of their paired ratings with the self-referent statement and conceptual markers related to intrinsic and extrinsic motivation, approach and avoidance behavior, and positive and negative valenced perceptions. Similarly, we grouped treatment descriptions related to side effects and calculated the mean of their paired ratings with the conceptual markers and the self-referent statement. A complete list of treatment descriptions is shown in Table 1. Independent samples t-tests were conducted to determine differences in ratings between participants with optimal and suboptimal adherence. The significance level for all statistical tests was set at 95% (a = .05).

Participant ratings of treatment-related items were also analyzed using ALSCAL, an MDS program. To simplify interpretation and describe how MDS could be applied in clinical work with HIV seropositive patients, we generated a two-dimensional configuration for two randomly-selected participants in this study. Brief interviews were conducted with participants following the study, and we present information from these interviews to describe how the configurations could be applied in clinical interventions. The MDS configurations selected for examination in this study had acceptable goodness-of-fit measures, with stress values ranging from 0 to 0.15.

## 3. Results

### 3.1 Sample Description

The sample consisted of African American men who described their sexual orientation as gay, bisexual, or with other terms that indicate MSM behavior. The mean age for participants in this study was 38 years (SD = 10.0). The bulk of the sample had annual incomes below #10,000 (71%; n =10). While the vast majority of participants reported receiving a high school diploma or its equivalent (79%; n = 11), only two participants indicated that they had earned a bachelor’s degree or a graduate level degree. The sample was evenly divided between participants who reported optimal adherence rates of 95% or greater and those who reported having adherence below those rates. The mean adherence rate was 79.4% (SD = 24.4). Table 2 provides a breakdown of participant characteristics by adherence level.

**Table 2:** Characteristics of participants by adherence level

Characteristic	All participants (n=14)	Optimal Adherence (n=7)	Suboptimal Adherence (n=7)	Test Statistic*	p**
Demographics					
Age, years	38±10.0	41±5.7	35±12.8	18.5	0.46
Adherence (M)	68.62% (SD=42.8)	96.4% (SD=9.45)	62.5% (SD=22.9)	2.5	0.02
Time since diagnosis (M, yrs.)	8.0 (SD=8.2)	10.8 (SD = 9.8)	5.1 (SD=5.5)	19.5	0.54
Depressive symptoms	31.2 (SD=9.6)	28.9 (8.8)	33.6 (10.5)	29.0	0.62

* Test statistics listed consist of t scores for continuous variables (age, adherence, and treatment duration); χ^2^ for categorical variables

** *p* values determined by Mann-Whitney U tests.

### 3.2 Paired Comparisons: Ratings of Treatment Goals

Participants completed all pairwise combinations of descriptions of their treatment goals to one another as well as the degree to which each description was associated with the following conceptual markers: 1) intrinsic and extrinsic motivation; 2) avoidance and approach-oriented behavior; 3) positively- and negatively-valenced responses; and 4) concrete or abstract identification. These conceptual markers were used to assess underlying views of treatment goals and their relationship to other implicit goals or needs (see Table 1).

Using a 7-point rating scale, participants with optimal adherence indicated that on the average, treatment descriptions related to side effects represented the way they usually thought about their treatment goal to a greater extent than those with suboptimal adherence (Ms = 3.5. vs. 1.7; p = .002). In addition, participants with optimal adherence were more likely to associate experiencing side effects with sources of intrinsic motivation (Ms = 3.4 vs. 1.9; p = .03). There were no other significant differences between optimal and suboptimal participants in terms of treatment framing based on similarity ratings of treatment descriptions and conceptual markers.

### 3.3 Multidimensional Scaling: Ratings of Treatment-Related Goals among Participants Based on Adherence

We conducted multidimensional scaling analyses of similarity ratings from the paired comparisons procedure to further explore the underlying relationship between patient treatment goals and other goals and needs. The MDS configurations generated in this study consist of two dimensions which represent how study participants thought about their treatment regimens, and how these thoughts influenced their treatment-related behavior. Dimensions were determined based on the proximity of treatment description items to each other and the conceptual markers. The clustering of treatment description items around conceptual markers indicates the extent to which participants perceived their similarity. As outlined in the following two cases, the configurations could be used to assess for information related to individual cognitive processes that either facilitate or impede adherence.

*Case 1: Patient with suboptimal adherence*. The participant was a 29-year-old male who had been diagnosed with HIV nearly two years before his enrollment in the study. He indicated that he had been in treatment for most of that time (20 months). His regimen included a single antiretroviral medication, which was to be taken once a day. At the time of his study enrollment, he reported that he had missed two of his doses during the past four days; for this reason, he was classified as having suboptimal adherence. With a score of 35, the participant’s depressive symptoms were high as assessed by the CES-D and far above the traditional clinical cutoff of 16 for the measure.

**Figure 1:**
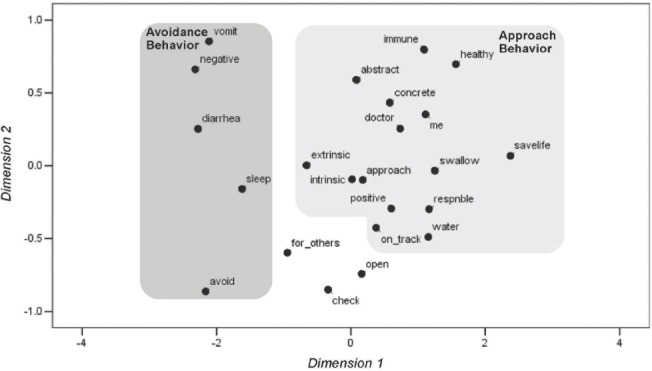
Multidimensional scaling map of stimulus items for Case 1. (Dimension 1 [avoidance/approach] vs. Dimension 2 [health/intrinsic and extrinsic].)

The first dimension in the participant’s MDS configuration, represented on the horizontal axis, is anchored by treatment descriptions and conceptual markers that differ in terms of their proximity to markers for avoidance and approach-oriented behaviors (see Figure 1).

Items depicted on the left side of this dimension include those related to treatment descriptions that spur avoidance (e.g., experiencing medication side effects) while those on the right side relate to approach-oriented descriptions of treatment (e.g., “keeping my life on track”). The second dimension of the configuration, as depicted on the vertical axis, is anchored by treatment descriptions related to health-related concerns and motivation type. Treatment descriptions associated with the health concerns, such as those related to side effects, are shown in the upper half of the configuration while those perceived by the participant as associated with either intrinsic or extrinsic motivation (e.g., “being responsible”) are depicted in the lower half. The clustering of items close to one another, particularly the conceptual markers indicates how these items were perceived by the participant.

The proximity of the participant’s self-referent “me” statement to the approach-oriented conceptual marker in his MDS map indicates that he focuses on approach-oriented treatment descriptions to a greater extent than those related to avoidance-oriented motivation for treatment. However, the positioning of some items in the participant’s map indicates his mixed views or ambivalence about treatment. For example, as indicated by his map, some treatment descriptions that are in close proximity to the marker denoting avoidance-oriented treatment behaviors also hold positive valences for the participant (e.g. “taking the medicine for my family, partner, or someone else in my life”). These contradictory views of treatment, as revealed by the MDS map, could be explored with such a patient in a clinical setting. This would be particularly helpful in cases where family members and significant others play a critical role in the patient’s health decisions. An MDS map could provide a way of identifying barriers to adherence that would otherwise be missed.

*Case 2: Patient with optimal adherence*. The participant was a 34 year-old male who had been diagnosed as HIV seropositive slightly more than four years prior to his study enrollment. He had been undergoing antiretroviral medications for the three years. At the time of the study, he was on his second antiretroviral regimen, which consisted of three medications. The participant reported that he had not missed any doses of his medications during the previous four days. The participant exhibited elevated levels of depressive symptoms with a CES-D score of 23.

**Figure 2:**
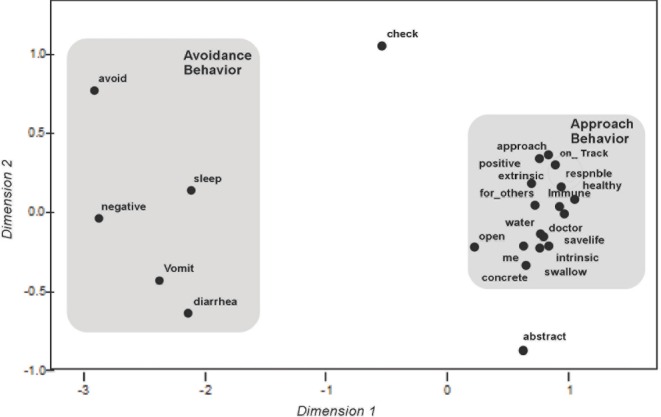
Multidimensional scaling map of stimulus items for Case 2. (Dimension 1 [avoidance/approach] vs. Dimension 2 [extrinsic/intrinsic].)

The participant’s MDS configuration is shown in Figure 2. The clustering of items depicted in his configuration differs from the mapping of the participant with suboptimal adherence. As the configuration illustrates, most items appear on the right of the configuration and are in close proximity to conceptual markers for approach-oriented behavior and positively valenced views of treatment. Furthermore, in contrast to the map for the participant with suboptimal adherence, most items for this participant are relatively close to the conceptual marker for intrinsic motivation. Maps for both participants indicate that “following doctor’s orders” is an approach-oriented description of treatment based on the proximity of the treatment description and the conceptual marker. For the participant with optimal adherence, however, the description of treatment as “taking the medicine for my family, partner, or someone else in my life” was viewed in positive and approach-oriented terms. In contrast to the first participant, this treatment description was viewed as having very little similarity to a thought about treatment that would spur avoidance-oriented behavior. During an interview following his completion of the paired comparisons procedure, the participant stated that he believes this was due to his cultural background and upbringing which valued interpersonal connections with members of his social network, particularly his family and community. The MDS map for this participant serves as an example of how these configurations could provide information about client strengths and cognitions that may facilitate treatment adherence.

## 4. Discussion

Recognizing the potential of paired comparisons and multidimensional scaling, several researchers have called for the application of these assessment and analytic tools to address clinical concerns (Darcy, Lee, & Tracey, 2004; Fitzgerald & Hubert, 1987; Houston, McKirnan, Cervone, Johnson, & Sandfort, 2011; Jaworska, Chupetlovska-Anastasova, 2009; Lease, McFall, Treat, & Viken, 2003). This preliminary study represents one of the first to explore the use of these tools in clinical interventions that would be aimed at promoting consistent adherence among patients with chronic illness and symptoms of major depression. The study illustrated how these analytic techniques could be used to assess patients’ underlying perceptions of their treatment goals in addition to mapping the relationship of the treatment goals with other personal goals, needs, and demands. In this way, findings presented in this exploratory report suggest that paired comparisons and MDS could be used to facilitate a patient-centered approach to health goal setting, such as one outlined by structural dynamics.

A structural dynamics approach views health and treatment goals as positioned within a broader mapping of the patient’s life goals, personal needs, values, and environmental demands or pressures (Shah & Kruglanski, 2008). The current study showed that participants with optimal adherence tended to view their treatment goals as associated with sources of intrinsic motivation. Furthermore, the study found that participants with optimal adherence were more likely to relate descriptions of treatment in terms of side effects in connection with intrinsic motivation. This finding suggests that patients with chronic illness and depressive symptoms may be aided in attaining favorable levels of adherence by connecting treatment, including aversive side effects, to goals and needs that are viewed as intrinsically motivating. For example, while some patients may view treatment behaviors as something that leads to side effects, they may also describe these treatment behaviors as linked to fulfilling family responsibilities, a potential source of intrinsic motivation. Such appeared to be the case based on the MDS configuration generated for a patient in this study with optimal adherence (Case 2). In contrast, the MDS configuration of the participant with suboptimal adherence suggested that this way of describing treatment triggered feelings of ambivalence (Case 1). As his map shows, this treatment description was in close proximity to both the markers for avoidance-oriented behavior and positive valence. In cases where family and others play a critical role in the patient’s life, this perception of treatment may be an area to address when designing a patient-centered intervention due to the potential conflicts it could pose.

### 4.1 Limitations

There are several limitations of this preliminary study that deserve mention. These limitations include a relatively small sample size that prevents generalizability of findings and a cross-sectional design that bars any conclusions related to causality. Differences between the two study groups in terms of age may also represent a limitation in that adaptive coping skills tend to increase with age. In addition, MDS configurations suggest that participants may not have understood the wording used for conceptual markers designed to access abstract and concrete views of treatment. These markers tended to appear in close proximity to one another, suggesting that participants viewed them as relatively similar. Future research should be conducted to address these limitations by, for example, using larger sample sizes and longitudinal designs to improve generalizability and address group differences. Despite the shortcomings of this exploratory study, our findings provide support for a role by MDS and paired comparisons in clinical interventions, particularly those designed to strengthen health-related goal setting with a structural dynamics approach.

## 5. Conclusion

Research shows that many patients with depressive symptoms experience difficulty in identifying their broader goals and using them to determine adaptive ways of thinking about or framing treatment-related behaviors (Leventhal, Diefenbach, & Leventhal, 1992; Valente, 2003; Watkins, 2011). An aim of clinical interventions using patient-centered approaches could include aiding patients in navigating their treatment goals by becoming aware of the multiple ways in which their health and treatment goals could be viewed, and how these goals are intertwined with other important goals and needs. The MDS configurations presented in this study provided patient-centered assessments of underlying cognitive processes that could be used in clinical interventions. For example, cognitive processes revealed in a patient’s MDS configuration that are related to treatment avoidance could be addressed by developing “if-then” implementation intentions (Sheeran, Gollwitzer, & Bargh, 2013). An individual patient might use these thoughts or cognitions to develop a personalized if-then plan geared toward improving their adherence goal: “If I find myself thinking about how I became infected with HIV, then I will direct all my energy and attention to thinking about staying healthy for my children.” The first part of the statement represents an avoidance-oriented cognition whereas the second part contains an approach-oriented one. In this way, information derived from such an assessment using the analytic tools employed in this study could be used as the springboard for clinical interventions. MDS could also be used to pinpoint specific areas of ambivalence and treatment-related views that could serve as barriers to consistent engagement with treatment goals.
